# Management of streptococcal pneumoniae-induced hemolytic uremic syndrome: a case report

**DOI:** 10.5414/CNCS107887

**Published:** 2014-04-14

**Authors:** Lauren Weintraub, Manpreet Ahluwalia, Samriti Dogra, Joan Uehlinger, Amy Skversky, Ljiljana Vasovic

**Affiliations:** 1 Pediatric hematology/oncology,; 2 Pediatric Nephrology, The Children’s Hospital at Montefiore, and; 3 Pathology, Montefiore Medical Center, Bronx, NY, USA

**Keywords:** hemolytic uremic syndrome, acute kidney injury, microangiopathic hemolytic anemia, thrombocytopenia, plasma exchange

## Abstract

Hemolytic uremic syndrome (HUS) secondary to *Streptococcus pneumoniae* infections (pHUS) has been well reported in the literature and accounts for roughly 5% of all the cases of HUS. However, this condition is likely under-diagnosed and the incidence is believed to be increasing. Given this increase in incidence of pHUS, it is important to have an understanding of the optimal means to manage the disease. We report a case of a 2-year-old male with pneumonia, acute kidney injury (AKI), microangiopathic hemolytic anemia (MAHA), and thrombocytopenia, diagnosed with pHUS and successfully treated with antibiotics, washed red blood cell (RBC) transfusions, plasma exchange (PE) with 5% albumin replacement, steroids, and hemodialysis. The response seen in our patient adds to the current literature and further supports the use of PE with albumin in patients with pHUS.

## Introduction 

Hemolytic uremic syndrome (HUS) is characterized by the triad of acute kidney injury (AKI), microangiopathic hemolytic anemia (MAHA), and thrombocytopenia. The most common presentation of HUS is preceded by a diarrhea prodrome, typically secondary to *Escherichia coli* O157:H7 [1]. However, HUS is also known to be caused by infection with *Streptococcal pneumoniae*. Streptococcal-induced hemolytic uremic syndrome (pHUS) accounts for ~ 5% of all HUS cases, however, the incidence is thought to be increasing in recent years [2, 3, 4]. Patients with pHUS typically present with either meningitis or pneumonia before progressing to MAHA, thrombocytopenia, and AKI. The pathophysiology of pHUS, however, differs from diarrhea associated HUS, by the presence of the Thomsen-Friedenreich or T-antigen. The pathogenesis of pHUS is attributed to an enzyme neuraminidase, which is produced by the *S. pneumoniae* bacteria. It cleaves the N-acetylneuraminic acid residues on the surface of red blood cells, glomerular endothelial cells, and platelets, thereby exposing the hidden T-antigen. Anti-T IgM antibody found in normal plasma may then bind the exposed T-antigens directly, causing AKI and thrombocytopenia [5]. The hemolytic anemia is likely the result of the microangiopathy, enhanced clearance of T-activated RBCs and possibly other non-immune mechanisms, but not interaction between anti-T and activated T-antigens on RBCs. Despite all that is known about this disease, there is no consensus in the literature on the best way to manage these patients. In review of the literature, we found one case series and two prior case reports detailing the successful treatment of pHUS with plasmapheresis [6, 7, 8]. However, there remains a lack of evidence to determine the definitive treatment for this condition and, to date, there are no randomized controlled trials. 

We describe a case of pHUS in a 2-year-old male with invasive pneumococcal disease and findings consistent with T-antigen activation that was successfully treated with PE. We hope to add to the existing literature on the use of PE in pHUS and further support the need for randomized control trials. 

## Case presentation 

### Initial presentation 

A 2-year-old previously healthy male of Albanian decent presented to the emergency room with cough. He was diagnosed with a viral upper respiratory tract infection and discharged home. Two days later, he returned to the emergency room with a worsening cough, productive of white sputum, shortness of breath, and fever. On review of systems, he was also noted to have decreased oral intake and several episodes of emesis. There were no known sick contacts. On exam, he was febrile, tachycardic, and tachypneic. He was noted to be pale and mildly dehydrated. Decreased breath sounds were appreciated over the right lung field. Initial labs were as follows: hemoglobin of 8.7 g/dL, hematocrit 28.7%, platelet count 474,000 g/dL, sodium 134 meq/L, potassium 3.8 meq/L, chloride 98 meq/L, HCO_3_ 15 meq/L, BUN 21 mg/dL, creatinine 0.5 mg/dL, glucose 67 mg/dL, calcium 9.3 mg/dL. His urinalysis showed specific gravity 1.016, trace ketones, 30 mg/dL protein, trace blood, and urine microscopy showed 4 WBCs and 2 RBCs. Initial chest X-ray showed a right sided middle and lower lobe pneumonia and a small right sided pleural effusion. In the emergency room, the patient was given a dose of ceftriaxone, started on oxygen for increased work of breathing, given an IV fluid bolus of normal saline and admitted to the general pediatric floor. 

His past medical history was significant for iron deficiency anemia diagnosed at 11 months of age; he had a normal hemoglobin electrophoresis at that time. Family history and social history was non-contributory. The patient has no known drug allergies. He was noted to have normal growth and development. 

### Hospital course 

On hospital day 3, the patient was noted to have increasing respiratory distress. Repeat chest X-ray and a chest sonogram showed a large right sided pleural effusion with multiple thick septations. He was treated with vancomycin and ceftriaxone for management of empyema. Concurrently, his BUN and creatinine increased to 67 mg/dL and 1.6 mg/dL, respectively, and his hemoglobin and platelet counts both decreased to 3.2 g/dL and 23 × 10^3^/µL, respectively. Lactic dehydrogenase (LDH) was 5,850 U/L, total bilirubin 2.1 mg/dL and direct bilirubin 0.8 mg/dL. The partial thromboplastin time (PTT) was prolonged to 60.7 seconds. The patient was transferred to the PICU for further care. 

Pediatric hematology, nephrology, and transfusion medicine were consulted. The peripheral smear was notable for toxic granulation in the neutrophils, large platelets, burr cells, target cells, microspherocytes, and schistocytes. Antibody screen was negative but the direct antiglobulin test was positive with both IgG and polyspecific antisera. Anti-I was identified at 4 °C. The patient’s RBCs mixed with 5 randomly selected, AB0-identical, antibody screen negative, normal adult serum samples showed strong agglutination (4+). This is consistent with T-antigen activation. Lectin agglutination was not done. Analysis for other etiologies of atypical HUS, including testing for Factor H and CH50, were not sent as these studies are not readily available at our institution. ADAMST13 testing was not performed as there was a low clinical suspicion for thrombotic thrombocytopenic purpura. 

Due to urgent transfusion need, washed RBCs were not initially available and the patient was given a RBC transfusion with a resultant increase in the hemoglobin from 3.2 g/dL to 5.5 g/dL. Forced-air warming was initiated in an attempt to keep the patient warm. 

On hospital day 4, he became oliguric. This coincided with a peak in his BUN and creatinine to 103 mg/dL and 2.8 mg/dL, respectively. A temporary hemodialysis catheter was placed and continuous veno venous hemodiafiltration (CVVHDF) was initiated. A right sided chest tube was placed. Blood cultures and pleural fluid cultures taken at two separate times during the hospitalization were negative, but the urine *Streptococcus pneumoniae* antigen was positive. His hemoglobin again dropped to 3.7 g/dL. At this time, he was transfused with washed RBCs. The hemoglobin was noted to increase to 8.4 g/dL and the platelet count to 22 × 10^3^/µL. Despite this increase, the patient had persistent hemolysis, and the hemoglobin again decreased to a low of 5.6 g/dL by hospital day 5. He was given an additional washed RBC transfusion with an increase in hemoglobin to 10.1 g/dL and an increase in platelet count from 18 × 10^3^/µL to 32 × 10^3^/µL. 

On hospital day 6, it was decided to initiate plasmapheresis secondary to pHUS disease progression. The patient weighed 14 kg and was 101 cm tall. The total blood volume was calculated as 1,442 mL. Because of the patient’s size, washed RBCs were used to prime the COBE^®^ Spectra for the PE procedure. The apheresis machine was primed according to the manufacturer’s instructions with normal saline and anticoagulant citrate dextrose A (ACD-A) and subsequently primed a second time with 0.5 U of RBCs. 5% albumin (~ 1 L per procedure) was used as replacement fluid. The ACD-A to whole blood ratio was 1 : 15. The access was a right internal jugular Shiley catheter. After the first PE, the hemoglobin increased to a high of 12.1 g/dL and then gradually decreased to 7.6 g/dL by hospital day 9. The platelet count gradually increased from 32 × 10^3^/µL immediately following PE, to 68 × 10^3^/µL by hospital day 9. At this time, it was decided to perform a second PE using the same prime and replacement fluid. The following day, the hemoglobin rose to 9 g/dL, and by hospital day 11, the platelet count increased to 166 × 10^3^/µL. 

**Figure 1.**
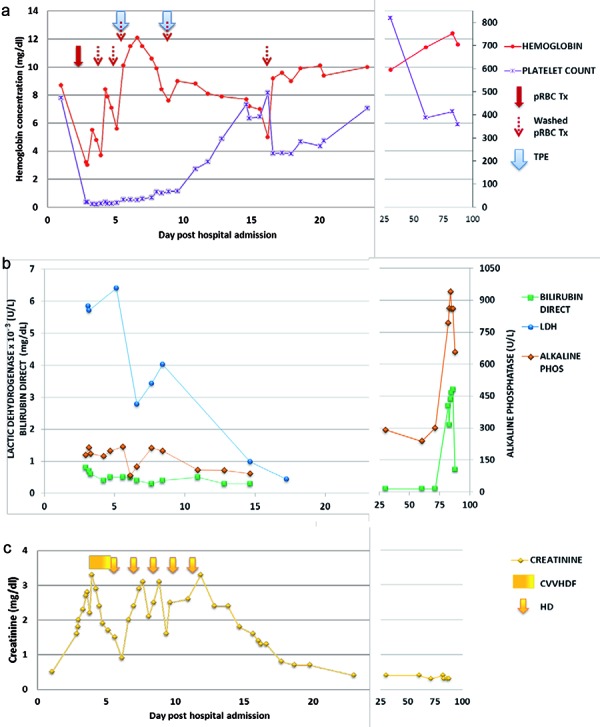
a: Platelet count and hemoglobin in relation to blood products and plasma exchange; b: LDH, bilirubin direct and alkaline phosphatase; c: Creatinine in relation to HD.

The hemoglobin gradually decreased following the second PE to 5 g/dL on hospital day 16. The patient was transfused with washed RBCs, after which the hemoglobin remained greater than 9 g/dL until discharge on hospital day 24. The platelet count remained stable from hospital day 11 until discharge. On the day of discharge, the hemoglobin and platelet count were 10 g/dL and 429 × 10^3^/µL, respectively. The two values remained normal on follow-up (Figure 1). 

With respect to his renal course, the patient was transitioned to intermittent hemodialysis on hospital day 6. He received a total of five hemodialysis treatments, his last treatment on hospital day 12. His creatinine on hospital day 12 was 3.3 mg/dL and declined to 0.4 mg/dL on hospital day 24. He went home on amlodipine for hypertension. 

The chest tube was removed on hospital day 11. On hospital day 15, the patient had significant desaturations with a new oxygen requirement. A chest CT revealed a large air cavity representing a pneumatocele of the right lung base. His antibiotic regimen was changed to zosyn and vancomycin. On hospital day 16 he had a right thoracotomy and decortication and placement of two chest tubes. Chest tubes were removed by hospital day 18. The patient was discharged from the hospital 5 days later on augmentin to complete a 3 week course. 

Three months following initial diagnosis of pHUS, the patient developed cholelithiasis and chronic cholecystitis and underwent a laparoscopic cholecystectomy. He has, otherwise, had no complications during a 10 month follow-up period. 

## Discussion 

Hemolytic uremic syndrome (HUS) is characterized by the triad of AKI, microangiopathic hemolytic anemia (MAHA) and thrombocytopenia. HUS can further be classified as diarrhea associated HUS, or atypical HUS (aHUS). Infection causes 90% of cases of hemolytic uremic syndrome. Most cases of HUS are secondary to Shigatoxin-producing *Escherichia Coli* (STEC), most frequently due to serotype O157:H7 STEC-HUS, however other infectious etiologies have been reported [9, 10]. Pneumococcal-induced HUS accounts for ~ 5% of all HUS in children; and appears to be increasing in prevalence in recent years [6]. Pneumococcal induced HUS must be differentiated from aHUS, a disorder of the alternative complement pathway. Atypical HUS accounts for ~ 10% of pediatric HUS cases and carries a poor prognosis, with a 25% mortality rate and 50% developing end-stage renal failure [11, 12]. The workup for aHUS includes eliminating the possible diagnosis of infection-associated HUS, measuring serum complement protein levels and factor H autoantibodies, and performing complement genetic testing [13, 14]. 

Prior studies have also shown a higher rate of mortality, up to 36%, when HUS is associated with pneumococcal infection [15, 16]. Long-term morbidity is also more significant in pHUS than in STEC-induced HUS [17]. This fact clearly demonstrates the need for better treatment options. 

### Pneumococcal-induced HUS management 

Historically, 50 – 70% of all episodes of invasive pneumococcal infections were caused by seven serotypes. The rate of infection with the vaccine serotypes (4, 6B, 9V, 14, 18C, 19F, and 23F) has declined dramatically. However, since the introduction of PCV 7, the United States has seen the emergence of serotype 19A as the most important invasive pneumococcal strain in young children [18]. Three to 4 years after the introduction of the 7-valent pneumococcal conjugate vaccine, the rate of serotype 19A invasive pneumococcal disease in children younger than 5 years increased significantly from 2.6 to 6.5 cases per 100,000 [18]. In a study of patients with pneumococcal HUS at The Children’s Hospital of Philadelphia (CHOP), serotype 19A infections increased from 1% in the pre-vaccine era, to 20% in the post-vaccine era [19]. In a subsequent study published from CHOP, the authors found that out of 12 patients with pHUS for which serotype data were available, 8 (66%) had serotype 19A. Interestingly, all 6 (100%) of the confirmed infections diagnosed after the introduction of the 7-valent pneumococcal conjugate vaccine were caused by serotype 19A. All serotypes of *S. pneumoniae* have neuraminidase activity capable of unmasking the T-antigen, however, the authors speculate that 19A is a serotype that may exhibit increased neuraminidase activity. Therefore, serotype 19A may now be more frequently associated with pHUS as a result of replacement disease in the post-vaccine era [4]. 

We were unable to isolate pneumococcus in our patient. The estimated yield of blood cultures from patients with pneumonia is only 10 – 30% [20]. In one study, culture-negative HUS was defined by pneumonia, meningitis, or other invasive infection and either T-antigen activation (positive Coombs test result) or the absence of disseminated intravascular coagulation [4]. Our patient did, however, have a positive urine antigen test for pneumococcus. The clinical significance of this test has been debated [21]. However, a recent study found that the pneumococcal antigen detection assay had a high sensitivity; positive tests were found in 96% of children with bacteremia and 76% of children with lobar pneumonia. They found a false-positive rate of 15% among febrile children without identified pneumococcal infection. The investigators concluded that, although not ideal, this combination of sensitivity and specificity compares favorably with other available tests [22]. 

Pneumococcal sepsis can cause disseminated intravascular coagulation and MAHA and must be differentiated from pHUS. A positive Coombs test result is sensitive for pHUS, but the specificity is unclear [4]. A positive Coombs test result is found in up to 90% of cases of pHUS [23]. Therefore, testing for T-antigen activation may be a useful predictor for the development of pHUS after invasive pneumococcal disease [2]. In a small study of 36 patients with invasive pneumococcal disease, the sensitivity of T-activation was found to be 86% for pHUS or hemolytic anemia associated with pneumococcal infection; the specificity was 57% [24]. Predicting the risk for pHUS would allow avoidance of blood products containing plasma when the risk of transmission of anti-T antibodies is high [6]. 

From prior studies, we understand that the pneumococcal organism produces a circulating neuraminidase, which cleaves N-acetylneuraminic acid from the glycoproteins on the cell membrane of erythrocytes, platelets, and glomeruli [25]. Removing the acetylneuraminic acid pathologically exposes the Thomsen-Friedenreich antigen, or T-antigen. This antigen can then react with anti-T IgM antibody present in plasma [26, 27, 28]. Prior studies have shown that this T-antigen activation, and subsequent reaction with antibody, occurs more frequently in infants and children [29, 30]. 

In the past, AB0 typing was performed with human antisera (anti-A and anti-B). Normal human serum also contains anti-T, so T-antigen activation in the past was initially identified from discrepancies between the typing of patient cells and patient serum. More recently, these reagents are all monoclonal, so T-antigen activation is now recognized without further testing. 

Once our patient was suspected to have T-antigen activation, further use of plasma containing blood products was avoided. The patient was transfused washed red blood cells with a more significant increase in hemoglobin than with unwashed. This has been previously described in the literature and is due to the fact that washing RBCs removes the plasma containing antibodies directed against T-antigen. Avoiding plasma containing products in patients with T-activation has been supported by multiple clinical reports [30, 31, 32, 33]. In their study in critically ill infants with T-antigen activation, Williams et al. [30] reported that hemolysis occurred only in those patients with T-antigen activation who received plasma-containing blood products. Therefore, they suggest that the use of washed erythrocytes and non-plasma-containing blood products is the safest approach unless severe thrombocytopenia or coagulation abnormalities develop. 

**Figure 2. Figure2:**
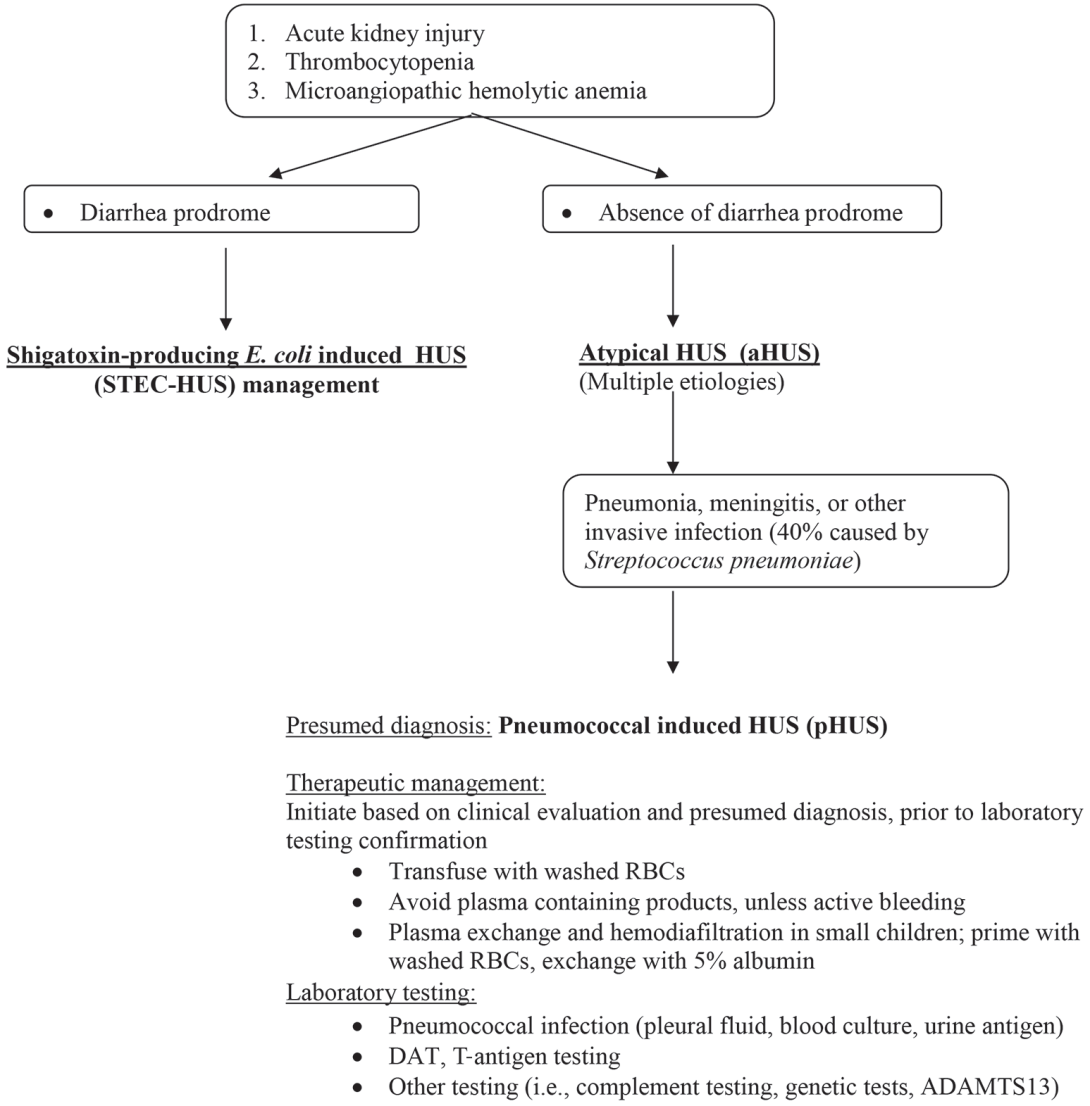
Algorithm for diagnosis and management of pHUS.

Additionally, our patient was found to have cold agglutinins consistent with anti-I. Therefore, forced-air warming was initiated in an attempt to keep the patient warm and prevent further agglutination. Also, it is known that anti-T is predominantly IgM and elicits a stronger in vitro reaction at 4 °C than at 37 °C [34, 35].[Fig Figure2]

Currently, there is insufficient evidence to either establish the efficacy or risk/benefit ratio of PE by the American Society for Apheresis [36]. It was suggested by Seger et al. [37] that PE may be able to reduce levels of circulating neuraminidase, and Beattie et al. [38] reported that PE reduces the circulating anti-T antibody titer. Recent reports suggest that PE represents a logical therapy for the management of pneumococcal induced HUS since PE has been demonstrated to reduce the level of circulating anti-T, as well as neuraminidase [6, 7, 8]. This would then limit the exposure of T-antigen on glomerular capillary endothelium and renal tubular epithelium and limit the reaction with anti-T [38, 39]. It is known that the anti-T is an IgM antibody that exists primarily in the intravascular space, and therefore would be efficiently removed by PE [40]. 

This use of PE for pneumococcal induced HUS has been inadequately studied in the literature. Prior reports have mentioned the use of PE with either plasma or albumin in pHUS cases, however the details of each case are not presented [3, 17]. In a paper documenting the experience of pHUS in the United Kingdom, Waters et al. [6], report the use of plasma exchange in 6 of 43 patients (16%). Replacement solution was documented for 5 of 6 patients. Albumin was used as replacement solution in 3 patients, and low-titer anti-T fresh frozen plasma (FFP) was used in 2 patients. One patient in the albumin group was noted to have chronic kidney disease on follow-up, and 1 patient in the FFP group had neurologic deficit with mild renal dysfunction. The authors concluded that replacement solution did not seem to have a significant impact on outcome in the patients who received FFP compared to the patients who received albumin. All 6 patients treated with PE survived. Overall mortality for all patients studied was 11% (5 patients). 

Hopkins et al. [7] and Petras et al. [8] detail the use of a PE in a 2-year-old and 4-year-old, respectively, with severe pHUS who both achieved a full recovery with PE and avoidance of plasma-containing blood components, including exchange with albumin. Our case report of a 2-year-old with severe pHUS, successfully treated with PE, further adds to the current literature. See Table 1 for summary of case reports.[Table Table2]

## Conclusion 

In summary, we have presented the case of a 2-year-old male with pHUS, diagnosed on the basis of invasive pneumococcal disease, *S. pneumoniae* antigen present in the urine, and T-activation, successfully treated with washed RBCs, PE with albumin and hemodialysis. Our study, in addition to the prior literature, suggests that PE contributes to complete recovery in these patients. We recommend that every patient with evidence of invasive pneumococcal disease, renal failure, MAHA and thrombocytopenia should undergo workup for pHUS, including evaluation for T-activation. It is essential to distinguish pHUS from aHUS given the difference in management. In pHUS, plasma containing blood products should be avoided and washed RBCs transfused based on a presumed diagnosis of pHUS and PE should be initiated if clinically warranted. See Figure 2 for diagnosis and treatment algorithm. 

## Conflicts of interest 

There are no conflicts of interest to declare. 

**Table 1.** Prior experience with PE in pHUS.

Study	Case	Identification of pHUS	Exchange	Outcome
Waters et al. 2007 [5]	43 patients with pHUS, ages 5 – 39 months, 6 treated with PE	Not specified (pneumococcus identified in 34 of 43 cases, T-activation identified in 36 of 37 cases	3 patients with albumin, 2 with FFP, 1 unknown	All survived, 1 patient in albumin group with chronic kidney disease (CKD), 1 patient in FFP group with neurologic deficit and mild CKD
Hopkins et al. 2008 [7]	2-year-old male	Blood culture positive for *S. pneumoniae*, T-activation	Albumin	Complete recovery, remained well at 7 month follow-up
Petras et al. 2012 [8]	4-year-old female	Chest tube drainage positive for *S. pneumoniae*, T-activation	Albumin	Complete recovery, no sequelae at 16 month follow-up
Our case	2-year-old male	Pneumonia, T-activation, urine antigen positive	Albumin	Complete recovery

**Table 2. Table2:** Summary of therapeutic interventions.

Hospital day	Symptom/Dx	Treatment
1 – 9	Fever, cough, SOB	Acetaminophen, Ibuprofen, O_2_, Ceftriaxone, Vancomycin
2 – 4	MAHA, Fe def. anemia	RBC transfusion, Ferrous sulfate
3 –	Oliguria, increased BUN/Cr	Continuous veno-venous hemodiafiltration (CVVHDF)
3 – 11	Empyema	Chest tube
4 – 16	MAHA, T-activation	Washed RBC, steroids
6 – 12	ARF	Intermittent hemodialysis (HD)
6 & 9	Worsening anemia and thrombocytopenia	Therapeutic plasma exchange (TPE) with albumin
16	O_2_ desat, pneumatocele	Right thoracotomy and decortication
78	Cholelithiasis	Cholecystectomy
